# Fatty Acid Profiles and Nutritional Evaluation of Fresh Sweet-Waxy Corn from Three Regions of China

**DOI:** 10.3390/foods11172636

**Published:** 2022-08-30

**Authors:** Ziqi Li, Tiannuo Hong, Zhenyu Zhao, Yanting Gu, Yanzhi Guo, Juan Han

**Affiliations:** 1 Institute of Food and Nutrition Development, Ministry of Agriculture and Rural Affairs, Beijing 100081, China; 2 Laboratory of Safety & Nutritional Function Risk Assessment for Agricultural Products of China Ministry of Agriculture and Rural Affairs, Beijing 100081, China

**Keywords:** fresh corn, fatty acid profile, sweet-waxy corn, fatty acids, nutritional evaluation, PCA, tocopherol

## Abstract

Fresh corn is a kind of herbaceous plant with rich nutritive value and a reasonable composition of fatty acids; however, there is little research on methods for the systemic nutritional evaluation of fatty acids in fresh corn. The aim of the present study was to conduct a comparative analysis of the fatty acid profiles of Chinese Huangnuo 9 sweet-waxy corn from the provinces of Inner Mongolia, Jilin, and Heilongjiang by gas chromatography; to establish a nutritional evaluation system according to the impacts of nutrients from fatty acids on human health; and finally, to evaluate, compare and rank fresh sweet-waxy corn grown in different regions. Tocopherols were detected by liquid chromatography in order to demonstrate the anti-oxidation activity of fresh corn’s fatty acids. The fatty acid contents and compositions of the 12 samples from the three regions are significantly different from each other. The nutrient value of the fatty acids in fresh corn was analyzed by factor analysis and a linear structural relation model, followed by the fitting and appraising of the model. The studied fresh sweet-waxy corn 1-4 from Inner Mongolia had the highest γ-tocopherol content and the closest saturated fatty acid:monounsaturated fatty acid:polyunsaturated fatty acid rate to the recommended value. The fatty acid profiles of sweet-waxy corns 2-1, 2-2, and 2-3 were the most diverse, and the comprehensive evaluation result of fresh corn 2-4 was the best; its total fatty acid content was the highest. Fresh corn 3-1 in Heilongjiang had the highest unsaturated fatty acids and lower values in its atherosclerosis index and thrombosis index, which suggested the strongest anti-atherosclerosis and anti-thrombotic ability. This work will give a reference to guide dietary choices and provide data support for dietary recommendations for residents.

## 1. Introduction

With the rise of problems such as obesity, cardiovascular disease, and cancer in humans in recent years, there has been increasing demand among consumers for high-quality food [1]. The composition of fatty acids in the diet determines the influences of lipid composition on human health [2,3,4], which is also closely related to chronic diseases such as diabetes and cardiovascular and cerebrovascular diseases [5]. Food fatty acid composition is of great significance to human nutrition and health issues. In addition, it also has an important position in various research areas, such as higher plant species classification [6]. Therefore, analyzing and evaluating food fatty acid composition is necessary in order to measure food quality [7].

Fresh corn is defined as corn picked from the late milk stage to the early dough stage by the Chinese national agricultural industry-standard NY/T523-2020 [8]. The nutritive value of fresh corn is better than that of dried corn because it is picked earlier than dried corn, and it is processed by vacuum and frozen in the stages after harvest. Fresh corn has the special properties of being fresh, sweet, soft, and waxy compared with ordinary corn, which is preferred by consumers all over the world. In addition, most of the nutrient contents in corn, such as amylopectin, soluble sugar, vitamin, crude fat, and protein contents, are higher than those in ordinary corn [9,10]. In recent years, with the in-depth study of food nutrition and the ideological changes in consumption and nutrition, what people know about fatty acids has increased; thus, consumers have turned more attention towards unsaturated fatty acids in plants [11,12]. Corn is considered to be a good source of unsaturated fatty acids.

Fresh corn is divided into three categories according to its variety, including sweet corn, waxy corn, and sweet-waxy corn. Sweet corn has the genetic characteristic of inhibiting soluble sugar conversion to starch; as such, it is rich in soluble sugar. Waxy corn tastes sweeter and stickier than ordinary corn, and has a lower content of soluble sugar than sweet corn. The soluble sugar content in waxy corn decreases faster than that in sweet corn at the filling stage, indicating that the soluble sugar in waxy corn is rapidly transformed into amylopectin at this stage [13]. Sweet-waxy corn is a new variety of corn bred by the crossbreeding method; it has the excellent characteristics and nutritional value of sweet corn and waxy corn. Through genetic hybridization, the same ear of sweet-waxy corn has both sweet and waxy grains, such that the corn has the sweetness and juiciness of sweet corn and the fragrance and softness of waxy corn. It is an emerging corn variety in recent years, is highly recognized among Chinese consumers, and has a high market share. At the same time, because of its rich nutritional value and special taste, sweet-waxy corn has huge development potential. Therefore, this study selects sweet-waxy corn from three provinces in the main producing areas of northern China as the object of research, comparison and evaluation, in order to establish a nutritional evaluation system for corn fatty acids, and their health effects on the population.

Lipids are one of the most important components of food [14], and the derivatives generated by the reaction of triglycerides and the oxidation products of fatty acids have certain biological activities which are closely related to the occurrence and development of atherosclerosis and premature aging [15,16]. Maize is composed of the seed coat, endosperm, and embryo; the embryo can be divided into the cotyledon, plumule, and radicle. The lipid content in the plumule is high, and the fatty acids in corn are concentrated in it. The lipids in corn are usually called healthy fats due to the influence of unsaturated fatty acids on blood cholesterol [17,18]. Therefore, corn can be consumed and can ingest a variety of unsaturated fatty acids, which can lower serum cholesterol and reduce the risk of atherosclerosis. In particular, ω-3 fatty acids can prevent colon cancer [19] and are also closely related to reducing the risk of chronic neurodegenerative disease [20], rheumatoid arthritis, osteoporosis [21], and kidney disease [22]. Detecting and analyzing the content and composition of fatty acids in corn can evaluate the nutritional quality of corn by the important index of fatty acids, can measure the nutritional value of maize, and can put forward suggestions for the balanced dietary intake of residents [23]. Unsaturated fatty acids have poor stability and are prone to peroxidation due to their unsaturated bonds. However, plant oils such as corn germ oil are rich in fat-soluble vitamin E (tocopherol), which can inhibit the peroxidation of unsaturated fatty acids by supplying phenolic hydroxyl to lipid free radicals [24], and are known as natural antioxidants. Hence, the composition and content of tocopherol are regarded as important indices to evaluate the quality of lipids.

A large number of foods rich in polyunsaturated fatty acids will lead to an increase in the number of neutrophils in the blood in a short period of time after their intake, thus increasing the inflammatory response [25]. Therefore, attention should be given to the intake of polyunsaturated fatty acids when eating fresh corn. Monounsaturated fatty acids (MUFAs) are a family of fatty acids that contain unsaturated bonds. Oleic acid is the most representative fatty acid in the regulation of blood lipids and the lowering of blood cholesterol. Its effect on lowering serum cholesterol and low-density lipoprotein is equivalent to that of linoleic acid [26], and oleic acid can also improve the immune response by interfering with macrophage lymphocytes and neutrophils [27]. Polyunsaturated fatty acids (PUFAs) refer to fatty acids containing two or more unsaturated bonds. The role of PUFAs in reducing blood cholesterol and protecting cardiovascular and cerebrovascular health is beyond doubt; therefore, the fatty acid analysis of MUFA and PUFA contents and the proportion of fatty acids for food quality research are of great significance.

There are few reports on the fatty acid analysis and evaluation of fresh corn, or even ordinary corn. The contents of fatty acids evaluated for fresh corn in China are mostly listed in one section of nutritional quality evaluation, and only the content of fatty acids is simply analyzed. The evaluation and analysis methods of fatty acids in other agricultural products are lacking, resulting in a lack of scientific analysis methods as the evaluation basis for the nutritional value of fatty acids in fresh corn. In this study, three provinces of China, referred to as Inner Mongolia, Jilin, and Heilongjiang, were selected as sampling areas, and the fresh sweet-waxy corn Huangnuo 9 was collected from the three regions. The fatty acid content and composition profiles of the fresh corn were detected by gas chromatography, and were analyzed by principal component analysis (PCA) and quality evaluation indices. The differences in fatty acid contents among the different regions were analyzed, and the reasons for the differences in fatty acid contents in sweet-waxy corn in different areas were further analyzed. The factor analysis of the principal component analysis (PCA) was carried out for different kinds of fatty acids in order to determine which fatty acids were the main factors in the nutritional quality of fresh sweet-waxy corn. Finally, according to the result of the factor analysis, the fatty acids in fresh sweet-waxy corn were evaluated comprehensively. In addition, the atherosclerosis index (AI), thrombosis index (TI), health promotion index (HPI), ideal fat index (DFA), and the fatty acid ratio of hypocholesterolemia to hypercholesterolemia (H/h) of sweet-waxy corn were calculated by physiological evaluation methods [28]; these indices could provide theoretical support for the nutritional evaluation of fresh corn. Except for the use of factor analysis to evaluate the comprehensive score, there are many indicators like natural antioxidants, such that the quality of fresh corn fatty acids needs to be combined with various fatty acid evaluation indices and the tocopherol content in order to comprehensively evaluate fresh corn.

A balanced diet has been one of the most important nutritive goals for Chinese residents in recent years [29]. The Chinese Society of Nutrition published the new “Dietary Guidelines for Chinese residents (2022)” on 26 April 2022. In this file, scholars proposed that Chinese people should eat more cereals and potato foods, and that they need to pay more attention to the balance of their diet. Fresh corn is a wholegrain food, and it can provide people with plenty of nutrition.

The main purpose of this study was to comprehensively analyze the fatty acid quality of fresh sweet-waxy corn based on fatty acid profiles and the content of tocopherol, in order to provide a reference for the establishment of a fatty acid evaluation system in the nutritional evaluation of fresh corn, and to provide a scientific basis for the dietary nutrient intake for the residents.

## 2. Materials and Methods

### 2.1. Reagents

The hydrochloric acid, ammonium hydroxide, pyrogallic acid, diethyl ether, petroleum ether (boiling range 30~60 °C), ethanol (95%), sodium hydroxide, boron trifluoride methanol solution (15%), anhydrous sodium sulfate, sodium chloride, sodium hydrogen sulfate, potassium hydroxide, absolute alcohol, ascorbic acid, amylase (activity ≥ 100 U/mg), and 2,6-butylated hydroxytoluene (BHT) were of analytical grade. The methyl alcohol, n-heptane, and isooctane were chromatographically pure. All of the above chemicals were purchased from Sinopharm Chemical Reagent Co. Ltd. (Shanghai, China). The triglyceride standard, mixed fatty acid methyl ester standard (purity ≥ 97%), 37 kinds of single fatty acid methyl ester standard, tocopherol (vitamin E) standards (purity ≥ 95%), and retinol standard (purity ≥ 95%) were obtained from Solarbio (Beijing, China). The distilled water and pH indicator paper were prepared by As One (Shanghai, China).

### 2.2. Plant Material and Sample Preparation

Fresh sweet-waxy corn Huangnuo 9 was collected from the Fuyuanquan Dongda ecological planting base in Wang Aizhao town, Dalad Banner, Erdos city, Inner Mongolia Autonomous Region, Suihua planting base in Heilongjiang Province, and Changchun planting base in Jilin Province. The corn was collected at the late milk stage of sweet-waxy corn, and the corn kernels were stored separately after threshing.

Fresh sweet-waxy corn products (including quick-frozen and vacuum products) were obtained from the Fengshuiliang Group in Dalad Banner, Erdos city, Jilin Xiangyu food company, and Heilongjiang Yuanye food company. The samples were divided into vacuum products and quick-frozen products, which were stored separately after threshing. The samples were ground using a laboratory homogenizer (MX16-waring-CB15K laboratory) purchased from Beyotime (Shanghai, China).

### 2.3. Quantification of Fatty Acids

#### 2.3.1. Solution Preparation

The fatty acid analysis was carried out by referring to the internal standard method of the first method in the Chinese National Food Safety Standard GB5009.168-2016 [30] and the method described by Danielli et al [31]. 

Then, 2.5 g undecarbonate triglyceride was placed in a beaker, and methyl alcohol was added to dissolve. The solution was transferred to a 500-mL volumetric flask; methyl alcohol was diluted to volume, and mixed. The mixed fatty acid methyl ester standard and 37 kinds of single fatty acid methyl ester standards were placed into a 10-mL volumetric flask, and n-heptane was added to dilute the reagent to volume; it was then stored in the refrigerator at −10 °C.

#### 2.3.2. Sample Pretreatment

Then, 2.0 mL triglyceride standard and 10 g of the corn sample were added to a 250-mL flat-bottom flask, and 100 mg pyrogallic acid, 2 mL ethanol (95%), and 4 mL distilled water were added and sufficiently mixed. Acid hydrolysis was selected as the method of sample hydrolysis. Then, 10 mL hydrochloric acid was added to the flat-bottom flask and shaken. The flask was heated in a water bath (70~80 °C) for 40 min. The sample was mixed every 10 min during the heating process. Then, the sample was cooled to room temperature after the reaction. In total, 10 mL ethanol (95%) was added to the sample after hydrolysis and shaking. The solution was transferred to a separatory funnel, and the flask was washed with 50 mL petroleum ether. Then, the bottles were shaken for 5 min and allowed to stand for 10 min. The above process was repeated three times. Eventually, the sample was collected into a 250-mL flask for rotatory evaporation until the solution was dry, and the remnant was the fat extract. Then, 8 mL sodium hydroxide and methanol mixed solution was added to the sample, the flask was connected to the reflux condenser, and a water bath (80 °C) was used to heat the sample until the oil disappeared. Then, 7 mL boron trifluoride methanol solution was added to the instrument and heated for 2 min (80 °C). The samples were cooled to room temperature. The sample was placed into the flask, and 7 mL n-heptane was added and mixed for 2 min. Then, the saturated sodium chloride solution was added to the flask, and the filtrate was stewed for stratification. Liquid supernatant was drawn for 5 mL into a 25-mL test tube, and 4 g anhydrous sodium sulfate was added, mixed for 1 min, and allowed to stand for 5 min. The supernatant was the final sample.

#### 2.3.3. Gas Chromatography (GC) Analysis for Fatty Acids

A 1.0 μL sample was injected and analysed by gas chromatography using a GC Agilent 7890B gas chromatograph (Agilent Technologies Inc. Ltd., Santa Clara, CA, USA) equipped with a hydrogen flame ionization detector and a TRACE TR-FAME fused silica capillary column (0.25 mm × 100 m, 0.25 μm; Thermo Fisher Scientific, Waltham, MA, USA). The initial column temperature was 100 °C, which was held for 13 min, followed by an increase to 180 °C at a rate of 10 °C min^−1^, which was held for 6 min. Then, the temperature was elevated to 200 °C at a rate of 1 °C min^−1^, held for 20 min, and finally raised to 230 °C at a rate of 4 °C min^−1^, which lasted for 10.5 min. The temperature of the injector was 270 °C, and the detector was 280 °C. The peak area was used for the quantitative analysis of the fatty acids.

The single fatty acid methyl ester(FAME) was calculated by the following formula:

$$X_{i}=F_{i}\times \left(A_{i}/A_{C11}\right)\times \left(\rho_{C11}\times V_{C11}\times 1.0067/m\right)\times 100$$

where *X_i_* denotes the content of FAME *i* (g/100 g), *F_i_* denotes the response factor of FAME *i*, *A_i_* denotes the peak area of FAME i, *ρ*_C11_ denotes the concentration of triglycerides (5.00 mg/mL), *V*_C11_ denotes the volume of triglycerides (mL), 1.0067 denotes the conversation coefficient of the undecarbonate triglyceride to methyl undecarbonate, and m denotes the quantity of the sample (mg).

The response factor of FAME *i* was calculated by the following formula:

$$F_{i}=\left(\rho_{si}\times A_{11}\right)/(\rho_{11}\times A_{si})$$

where *F_i_* denotes the response factor of FAME i, *ρ_si_* denotes the concentration of single FAME *i* in the mixed standard solution(mg/mL), *A*_11_ denotes the peak area of undecarbonate triglyceride, *A_si_* denotes the peak area of FAME *i*, and *ρ*_11_ denotes the concentration of undercarbonate triglyceride in the mixed standard solution(mg/mL).

#### 2.3.4. Quality Control

We took an equal number of 5, 10, 20 mg/mL standard liquids to the test tube, respectively, then compared the peak area of different concentrations in order to determine the accuracy of the instruments, recovery, LOD and LOQ (Table 1). Then, we took the consistence as the X-axis, the peak area as the Y-axis, and drew the standard curve. 

### 2.4. Quantification of Tocopherol

#### 2.4.1. Solution Preparation

The tocopherol analysis was carried out by referring to the first method in the Chinese National Food Safety Standard GB5009.82-2016 [32].

In total, 50 g potassium hydroxide was dissolved in 50 mL distilled water, and 200 mL petroleum ether and 200 mL ether were mixed. Then, 25 mg of the retinol standard was dissolved in absolute ethanol, transferred into a 50-mL volumetric flask, and brought to volume with absolute ethanol. In total, 50 mg of the tocopherol standard was placed into the beaker, and ethanol was added to dissolve it. Then, the solution was transferred into a 50-mL volumetric flask and brought to volume with ethanol. The solutions were heated to 20 °C, and the concentration was corrected before use. A total of 1 mL the above retinol standard solution and 5 mL the tocopherol standard solution were mixed into a 50-mL volumetric flask and then brought to volume with methanol. The two standard solutions and the mixed standard solution were stored in the shade at −20 °C. Then, 0.20 mL, 0.50 mL, 1.00 mL, 2.00 mL, 4.00 mL, and 6.00 mL were placed into 10 mL volumetric flasks and diluted with methanol to the volume.

#### 2.4.2. Sample Preparation

In total, 5 g of sample was placed into a 150-mL bottom flask, and 20 mL warm water was added to dissolve the powder. Then, 5 g amylase was added. The flask was oscillated at a constant temperature of 60 °C in the dark for 30 min. Then, 1 g ascorbic acid and 0.1 g BHT was added to the flask, and 30 mL absolute ethanol and 10 mL potassium hydroxide solution were added and mixed. Finally, the mixture was oscillated at a constant temperature of 80 °C for 30 min and cooled to room temperature with cool water. The sample and 50 mL of the petroleum ether and ether mixed solution were transferred into a 250-mL separatory funnel for extraction for 5 min, and the lower layer phase was collected and transferred to a 250-mL separatory funnel. The previous step was repeated, and the ether layer was collected. In total, 10 mL distilled water was used to wash the ether layer, which was repeated 3 times until the pH was near neutral. Then, the lower water phase was removed and discarded. The solution was filtered through 3 g anhydrous sodium sulfate to a 250-mL rotary evaporator, and the separatory funnel and anhydrous sodium sulfate were washed with 15 mL petroleum ether. Then, rotary evaporation was used to reduce the pressure distill in a 40 °C water bath, and the evaporator was removed when the solution reached 2 mL. The remains were washed with ether, removed into a 10 mL volumetric flask, and brought to volume; the solution was subjected to organic filtration (0.22 μm).

#### 2.4.3. Liquid Chromatography (LC) Analysis for Tocopherol

A 10 μL sample was injected and analysed by liquid chromatography using an Agilent 1290 Infinity II liquid chromatograph (Agilent Technologies Inc. Ltd., Santa Clara, CA, USA) equipped with a UV detector and an AcclAim C30 3 μm column (250 m × 4.6 mm × 3 μm; Thermo Fisher Scientific, Waltham, MA, USA). The column temperature was 20 °C, and the flow rate was 0.8 mL min^−1^. Distilled water was mobile phase A, and methanol was mobile phase B. The ultraviolet wavelength of tocopherol was 294 nm.

The content of tocopherol was calculated by the following formula:

X=(ρ×V×f×100)/m

where *X* denotes the content of tocopherol in the sample (mg/100 g), *ρ* denotes the concentration of the tocopherol calculated by the standard curve (μg/mL), *V* is the volume of the volumetric flask (10 mL), *f* denotes the conversion factor (0.001), and *m* denotes the quantity of the sample (5 g).

#### 2.4.4. Quality Control

As showed in Table 2, we added 1.0, 0.1, 0.4 and 0.05 mg/ ml of standard liquid to the test tube; we took the consistency as the X-axis, the peak area as the Y-axis, and drew the standard curve. 

### 2.5. Evaluation of Six Nutritive Indices

Fatty acids play an important role in human growth, development, and life activities, and different kinds of fatty acids have their corresponding physiological functions [33]. In this study, 6 evaluation indices of fatty acids commonly used in physiology and nutrition were selected for the nutritive evaluation of the fatty acids in fresh sweet-waxy corn.

#### 2.5.1. Fatty Acid Quality Index (FAQI)

Generally, fatty acid quality evaluation in food includes three indices, namely the atherosclerosis index (AI), the thrombosis index (TI), and the fatty acid ratio of hypocholesterolemia to hypercholesterolemia (h/H), which are collectively known as the fatty acid quality index (FAQI) [31].

(1) Atherosclerosis Index (AI) [34]

Atherosclerosis is a chronic progressive artery disease, and its causes are complex, including many factors, such as an abnormal nonspecific inflammatory response, the oxidative stress of lipid metabolism, and the proliferation of vascular smooth muscle cells [35]. An excessive intake of saturated fatty acids (SFAs) will increase serum cholesterol and have a high atherogenic effect, while an appropriate intake of ω-6 fatty acids is conducive to the prevention of atherosclerosis [34]. Therefore, one of the most important indicators for the evaluation of dietary fatty acids is the atherosclerosis index (AI) of food.

(1) AI=(C12:0+4×C14:0+C16:0)/(∑MUFA+∑PUFA) 

(2) Thrombosis Index (TI)

At present, there are few studies related to the food TI in China, and most of the foreign studies are in the field of milk and meat products. The nutritional value of different foods can be judged by calculating the thrombosis index of different food TI values. Similarly to the AI value, TI is an evaluation index of fatty acids proposed by Ulbricht in 1991; it can be used as a marker of potential platelet aggregation [36]. Many nutrition-related studies have shown that foods with lower AI and TI values have higher prevention potential for coronary heart disease [31,37], and the nutritional quality of such foods is also better.

(2) TI=(C14:0+C16:0+C18:0)/(0.5×∑MUFA+0.5×∑PUFA(n−6)+3×∑PUFA(n−3)+∑PUFA(n−6)/∑PUFA(n−3))  

(3) Fatty acid ratio of hypocholesterolemia to hypercholesterolemia (h/H)

The ratio of fatty acids between hypocholesterolemia and hypercholesterolemia is a concept integrated by Santos-Silva in 2002 [38]. By calculating the ratio of fatty acids that do not increase blood cholesterol after consumption and those that can increase blood cholesterol after consumption, we can evaluate whether a food is healthy and whether its nutritional value can be recognized by consumers.

(3) h/H=(C18:1 n−9 cis+∑PUFA)/(C12:0+C14:0+C16:0)

#### 2.5.2. Health Promotion Index (HPI)

The Health Promotion Index (HPI) refers to the inverse of the AI, which was proposed to describe the fatty acid quality of milk [39]. The higher the HPI is, the weaker the atherogenic ability of the food is and the more nutritious it is [28]. The HPI value was proposed in 2004, but it is rarely used in fatty acid evaluation except in milk due to its overlapping description with the AI value. However, the HPI value evaluation of milk products suggests that HPI may affect the taste and flavour of milk products, and unsaturated fatty acids are easily oxidized at high temperatures, which has adverse effects on flavour in the processing of fresh corn. Corn is cooked by cooking and vacuum or freezing, which is beneficial to the preservation of unsaturated fatty acids.

(4) HPI=UFA/(C12:0+4×C14:0+C16:0)

#### 2.5.3. Peroxidation Index (PI)

The peroxidation index represents the relationship between fatty acid composition and tissue oxidation sensitivity. The higher the PI value is, the higher the oxidation sensitivity of the food. Usually, the PI value is expressed as a percentage. Studies have shown that the PI value of meat decreases significantly after heat treatment [15], indicating that heat treatment can significantly promote the oxidation of unsaturated fat in food. In addition, the higher the PI value is, the greater the protective potential for coronary artery disease [40]; the calculation formula for this is as follows:

(5) PI=(0.025×Monooleic fatty acid)+(1×Diene fatty acid)+(2×Triene fatty acid)+(4×Tetraene fatty acid)+(6×Pentene fatty acid)+(8×Hexene fatty acid)  

#### 2.5.4. Nutritional Value Index (NVI) [41]

The NVI is used to describe the potential impact of different types of lipids on health [42]. Studies have shown that most of these indices are between 2 and 3 [43].

(6) HVI=(C18:0+C18:1)/C16:0

### 2.6. Statistical Analysis

Excel was used to process the original data, and the experimental data are expressed as the mean ± standard deviation of three parallel tests. SPSS 21.0 (IBM, Armonk, NY, USA) and GraphPad Prism 9.0 (GraphPad Software, San Diego, CA, USA) were used to compare the components of fatty acids in different areas in single factor variance analysis (ANOVA, San Francisco, CA, USA), and the means were compared by Duncan’s test at 0.05 significance. Principal component analysis was accomplished using SPSS 21.0. An extreme correlation is defined as a significant correlation between fatty acids at the 0.01 level.

The PCA index weight was calculated by the entropy method [44].

Y1 and Y2 are, respectively, functional expressions of principal components P1 and P2, respectively, and the feature vector X was obtained by dividing the corresponding value of the component matrix by the square root of the feature value. 

## 3. Results and Discussion

### 3.1. Fatty Acid Profiles and Characteristics

SPSS (IBM, Armonk, NY, USA) software was used for the ANOVA of the detected fatty acid data, which are marked in Table 3 and Table 4.

The fatty acid composition profile of fresh corn in the three regions is presented in Table 3. The 12 samples showed differences in fatty acid composition; seven kinds of fatty acids in fresh sweet-waxy corn 3-3 were detected, then fresh corn 1-3 showed 11 fatty acids, and fresh corns 1-5, 3-1, and 3-2 showed 12 fatty acids. Then, 13 fatty acids were detected in fresh corns 1-1, 1-2, and 1-4 from Inner Mongolia, and 14 kinds of fatty acids were detected in fresh corn 2-4. Fresh corns 2-1, 2-2 and 2-3 had the greatest variety of fatty acids (15 kinds of fatty acids). Fresh corn 2-4 in Jilin had the most fatty acids (1.99 ± 0.12 g/100 g). The total fatty acid content of the fresh corn was approximately 0.81 ± 0.00~1.93 ± 0.06 g/100 g. As we can see in Table 3, the content of linoleic acid (C18:2 n6c) in fresh corn was the highest, and its percentage of total fatty acids was 40.51 ± 1.12~61.25 ± 0.09%, followed by oleic acid (C18:1 n9c) at 20.48 ± 0.08~36.66 ± 0.12%. In addition, the content of palmitic acid (C16:0) was also high (13.48 ± 0.14~19.50 ± 0.12 %).

In Table 3, the fatty acid composition among the 12 samples was different. Capric acid (C10:0) and tricosanoic acid (C23:0) were only detected in fresh corns 2-1, 2-2, 2-3 and 2-4 from Jilin; eicosapentaenoic acid (EPA, C20:5 n3) was detected in fresh corns 1-1, 1-4 and 1-5 from Inner Mongolia. Lignoceric acid (C24:0) was detected in fresh corns 1-1, 2-1, 2-2 and 2-3.

In Table 4, the relative content of saturated fatty acids (SFAs) in fresh sweet-waxy corn was approximately 15.55 ± 0.22~22.52 ± 0.48%, and monounsaturated fatty acids (MUFAs) were approximately 21.05 ± 0.06~37.09 ± 0.08%. It was 41.72 ± 1.04~63.14 ± 0.00% in polyunsaturated fatty acids (PUFAs). Palmitic acid and stearic acid have the highest proportion (>95%) in SFAs, which is higher than that of seaweed, with 81% [1], and other grape plants, with 85~94% [45]. The values of SFA:MUFA:PUFA in fresh corn were around 1:1.2:2.5. The fatty acid ratio of fresh corn 2-1 was closest to the reasonable dietary fatty acid ratio of 1:1:1 [46], while the PUFA content in other samples was relatively high but still within the acceptable range. Therefore, the fatty acid composition of fresh corn was reasonable, making it a high-quality fatty acid food.

Linoleic acid and α-linolenic acid are essential fatty acids that cannot be synthesized in the human body and need to be absorbed through food [47]. Studies have shown that linoleic acid can promote metabolism, enhance lipolysis, and prevent atherosclerosis [48,49,50]. Linoleic acid could also improve insulin sensitivity; as such, it can also reduce the incidence of type 2 diabetes [17]. α-Linolenic acid can reduce blood lipids and improve liver and neurological function [51,52,53], and α-linolenic acid is the precursor of eicosapentaenoic acid (EPA), which is highly effective in preventing and treating heart disease [54]. According to the latest study, linoleic acid and linolenic acid can protect the liver and improve the abundance of intestinal flora in mice when ingested in an appropriate proportion [55]. The ratio of ω-3:ω-6 fatty acids in the three regions is shown in Table 4. The ratio of ω-6 fatty acids to ω-3 fatty acids in fresh sweet-waxy corn is between 16.04 ± 0.49 and 38.6 ± 0.77. According to the current Chinese society of nutrition, with regard to the dietary nutrient reference intake of Chinese residents, the recommended dietary ratio of ω-6:ω-3 fatty acids is (4~6):1, but there are differences in ω-6:ω-3 among different species [33]. Therefore, the fatty acid composition of fresh sweet-waxy corns 2-2, 2-3 and, 2-4 was more suitable for the intake of people lacking ω-6 series fatty acids. In addition, other studies have shown that the proportional intake of PUFAs has a comparable effect on neoplastic bone disease due to the antioxidant activity of polyunsaturated fatty acids [56]. In addition, the P/S recommended by the World Health Organization (WHO) was 0.4 [57], and the P/S of fresh corn in the three regions was between 2.2 and 3.6. Thus, fresh corn is a balanced dietary source of fatty acids. However, according to the viewpoints of some studies, the P/S value cannot be used as a scientific method to evaluate the quality of fatty acids alone, mainly because partial SFA does not increase plasma cholesterol content. In addition, the P/S value ignores the influence of MUFAs to some extent in this index [58]. Therefore, when evaluating dietary fatty acids, the P/S value should be combined with other indicators, such as the atherosclerosis index (AI) and the thrombosis index (TI). Regarding the ratio of SFAs to MUFAs, researchers generally believe that a high-MUFA and low-SFA diet can reduce blood cholesterol and low-density lipoprotein content [25]. The MUFA/SFA values in fresh corn in the three places were all over 1, indicating that fresh sweet-waxy corn has a good lipid-lowering ability.

### 3.2. Factor Analysis

The correlation coefficient matrix of each fatty acid’s content is shown in Table 5. The contents of palmitic acid and the contents of palmitoleic acid, stearic acid, oleic acid, linoleic acid, and arachidic acid had extremely significant positive correlations (*p* < 0.01). The palmitoleic acid content was extremely positively correlated with the content of stearic acid, oleic acid, and arachidonic acid (*p* < 0.01). There was an extremely significant positive correlation between stearic acid and arachidonic acid (*p* < 0.01). Oleic acid significantly correlated with linoleic acid (*p* < 0.05), and had an extremely significant correlation with arachidonic acid (*p* < 0.01). Cis-11-Eicosenoic acid was only correlated with linoleic acid (*p* < 0.01). 

Due to the correlation between different fatty acid contents, factor analysis can be used to evaluate the synthetic quality of fatty acids. Principal component analysis (PCA) was selected as the factor analysis method in this study. As described in Table 6, the cumulative variance contribution rate reached 88.327%, which can represent most information on the fatty acid composition in Huangnuo 9. Reducing this dimension can eliminate the relativity of the sample data. The maximum variance method was selected for data rotation processing in factor analysis in order to obtain the rotation component matrix (Table 7).

**Figure 1:**
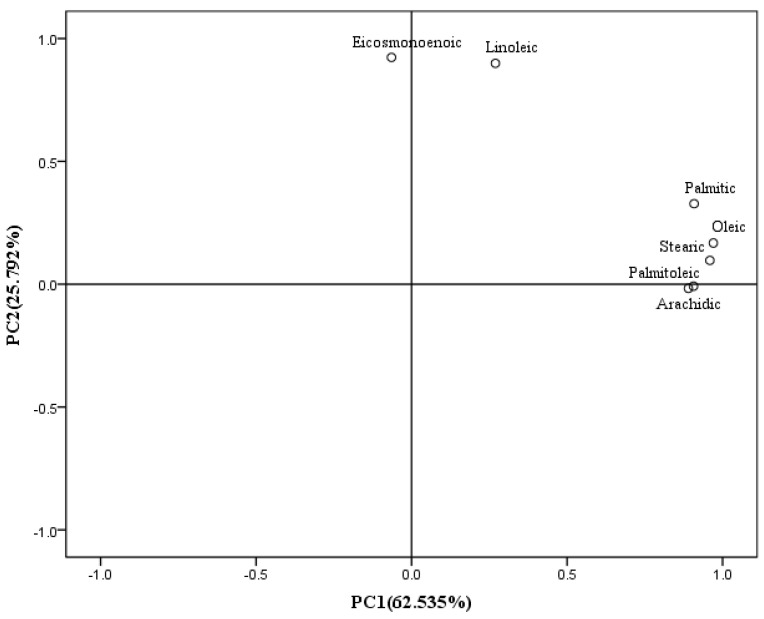
Rotation loading diagram of the fatty acid principal component analysis of fresh sweet and glutinous maize.

Figure 1 was the rotation loading diagram of fatty acids. Palmitic acid, palmitoleic acid, stearic acid, oleic acid and arachidonic acid had a large load in principal component 1, and the contribution rate of the rotation variance of principal component 1 (PC1) was 62.535%, indicating that these fatty acids had a great impact on the nutritional quality of fresh sweet-waxy corn. The variance contribution rate of PC2 reached 25.792%, and linoleic acid and cis-11-eicosenoic acid had large loads in PC2.

PCA was used to analyse 7 fatty acids in sweet-waxy corn, and the rotation component matrix of the fatty acid factor analysis is shown in Table 7. Oleic acid can reduce serum cholesterol and improve the immune response, while stearic acid has antiatherosclerotic properties [59]. According to the results of the factor analysis, fatty acids with relatively large loads in PC1 are mostly fatty acids that can resist atherosclerosis and reduce cardiovascular and cerebrovascular diseases; thus, PC1 was named as the cardiovascular disease decrease component principal. Linoleic acid and α-linolenic acid are essential fatty acids that have the largest load in PC2. Therefore, PC2 is characterized by essential PUFAs, and PC2 was named as the unsaturated fatty acid principal component.

The weight of each index was obtained by the weight factor decision method. The index weights of different fatty acids were calculated by combining the variance contribution rates of the two principal components (Table 8). As can be seen from Table 8, the most important fatty acids affecting the quality of fresh sweet-waxy corn were palmitic acid (0.169), oleic acid (0.158), and stearic acid (0.148), followed by linoleic acid (0.146). These four fatty acids accounted for more than 62% of the index weight of fresh corn, and had a great impact on the quality of the corn fatty acids. Therefore, in the evaluation of the fatty acids of fresh corn, more attention should be given to the four fatty acids according to the index weight, rather than only focusing on unsaturated fatty acids.

The Z score method was used to standardize the data. A comprehensive evaluation formula constructed according to the variance contribution rate results of the factor analysis was $F=0.708\times Y_{1}+0.292\times Y_{2}$. 

$Y_{1}=0.434\times Z_{1}+0.433\times Z_{2}+0.458\times Z_{3}+0.464\times Z_{4}+0.129\times Z_{5}+0.425\times Z_{6}-0.031\times Z_{7}$; $Y_{2}=0.243\times Z_{1}-0.006\times Z_{2}+0.072\times Z_{3}+0.125\times Z_{4}-0.669\times Z_{5}-0.013\times Z_{6}+0.687\times Z_{7}$.

Finally, the comprehensive evaluation score and ranking of fatty acid quality of fresh sweet and glutinous corn in the three places were obtained (Table 9).

As shown in Table 9, the highest fatty acid comprehensive score was that of fresh corn 2-4, followed by 2-2 and then 2-1. These three samples were all obtained from Jilin province; the reason for this is mainly due to the long history of fresh corn planting in northeast China and the germplasm resources in Jilin. It has good resources and rich experience in planting technology and environment, which is worth learning and understanding in the other main producing areas of fresh corn in north China [60].

### 3.3. Evaluation of Six Nutritive Indices

#### 3.3.1. Fatty Acid Quality Index (FAQI)

(1) Atherosclerosis Index (AI)

According to formula (1), the atherosclerosis index of fresh corn in the three regions can be calculated (Table 10), and the AI value of fresh corn in the three regions has a significant difference (*p* < 0.01), ranging from 0.21 ± 0.00 to 0.44 ± 0.03, which is lower than that of potato [61], Blackspot Sisord Catfish [62], cattle, sheep and pork [63], close to the value of fish [25]. There is no authoritative evaluation value for the index of atherosclerosis in food [61], but the high AI values may lead to the immune system and circulation system, and cell adhesion. A low AI value can prevent arteriole and large coronary artery disease according to the literature [64]; thus, the lower AI in food is associated with better health.

(2) Thrombosis Index (TI)

The TI calculated according to formula (2) is shown in Table 10. The TI of fresh sweet-waxy corn in the three places is 0.01 ± 0.00~0.02 ± 0.00, which is less than the bean TI value (0.10) [65]. AI and TI values are proposed mainly in consideration of the possible impact of single fatty acids on human health [66]. Therefore, the correlation between the two indicators in food is particularly important.

(3) Fatty acid ratio of hypocholesterolemia to hypercholesterolemia (h/H)

The h/H value was initially measured at between 1.8 and 2.1 [5,67]. Wood and Enser measured the h/H value of a pork chop in 1997 at approximately 2.08 [68]. In this experiment, the h/H value of sweet and glutinous corn in fresh food was approximately 3.29 ± 0.08~4.55 ± 0.00, far higher than the h/H value of livestock, poultry, fish, or raw milk [69], with higher nutritional value [70] and greater benefit to the human body.

#### 3.3.2. Health Promotion Index (HPI)

According to Formula (4), the HPI values of fresh sweet-waxy corn in the three places can be calculated. The results are shown in Table 10. The HPI values of fresh corn are 2.28 ± 0.13~4.72 ± 0.05, higher than the milk HPI value (0.359-0.435 [28]), indicating that fresh corn is rich in unsaturated fatty acids, tastes better, and has higher nutritional value. In addition, there were significant differences in the HPI values of fresh corn between the different regions (*p* < 0.01).

#### 3.3.3. Peroxidation Index (PI)

According to formula (5), the PI value of fresh corn was approximately 0.50 ± 0.01 to 1.15 ± 0.07 (Table 10). It should be noted that a high PI value also means a high oxidation sensitivity of fresh corn, which needs to be considered during processing, storage, and consumption.

#### 3.3.4. Nutritional Value Index (NVI)

According to formula (6), the nutritional value index (HVI) of fresh corn is approximately 1.13 ± 0.00~2.29 ± 0.03 (Table 10). Generally speaking, the higher the value is, the more the octadecarboxylic fatty acids are relatively suitable for consumption.

Here, we evaluated and analyzed the nutritional quality of fatty acids in fresh corn by analyzing the composition and content of fatty acids in fresh sweet-waxy corn, and we calculated six evaluation indices of fatty acids. In terms of fatty acid composition, there were differences in the fatty acid composition and content among fresh corn, but the composition and content of fatty acids in the same regions were relatively close, and the differences were relatively small. In total, 15 fatty acids were detected in fresh maizes 2-1, 2-2 and 2-3 from the Jilin region, and the composition of fatty acids was the most diverse. The UFAs accounted for 78.03 ± 1.06~84.45 ± 0.22% of the total fatty acids. Studies have shown that α-linolenic acid has a variety of biological activities [71], such as the prevention and treatment of heart disease [72]. In addition, it is easy to isomerize and form conjugated fatty acids under high-temperature and alkaline conditions [73]. Compared with ω-6 fatty acids, it has a higher nutritive value and is more beneficial to the human body.

After the evaluation of the physiological and nutritional indices of fresh corn, it can be seen that the AI value and TI value of fresh corn in the three places are lower than those of other agricultural products, which has little effect on atherosclerosis and thrombosis and is more suitable for people of all ages to eat. The fatty acid PI value of maize in the three regions is higher, and it is highly sensitive to oxidation and has a strong ability to protect against coronary artery disease. Therefore, attention should be given to the control and optimization of oxidative damage in maize during processing and storage. The h/H value of fresh corn in the three regions was significantly higher than that of meat and raw milk, indicating that the fatty acid content of fresh corn with hypocholesterolemia was higher than that of hypercholesterolemia, which had a significant preventive effect on human cardiovascular diseases. The HVI value can describe the nutritional value of octadecanoic fatty acids, and its value depends on the contents of stearic oleic acid and palmitic acid. In fresh corn, the HVI value is between 1 and 3, indicating that the content of palmitic acid is slightly lower than the sum of stearic acid and oleic acid in fresh corn.

According to the six nutritional evaluation indices of fresh corn, fresh sweet-waxy corn had the characteristics of a low atherosclerosis index, low thrombosis index, and high peroxidation index. The total fatty acid content of maize is higher than that of other main grains and is rich in unsaturated fatty acids (UFAs), so the nutritional value of maize fatty acids is higher, which is worth further development and utilization. AI and TI values of fresh corn 2-4 are low, and the results show that the atherogenic and thrombogenic properties were weak, but its tocopherol content was not high.

### 3.4. Vitamin E Content Analysis

Maize is rich in lipids, which provides a good environment for the retention of fat-soluble fatty acids. Vitamin E (tocopherol), as a good natural antioxidant, plays an important role in the nutritional composition of maize and the nutritional evaluation of fatty acids [74]. The results showed that when the content was less than 100 mg/100 g [75], the antioxidant activity of γ-tocopherol in vitro was better than that of α-tocopherol and δ-tocopherol [76]. In addition, γ-tocopherol has been proven to inhibit and promote cancer or increase the expression of tumour suppressor genes by interfering with the signaling pathway of cell cancer [77]. Through detection, vitamin E mainly exists in the form of α-tocopherol, γ-tocopherol and δ-tocopherol in fresh corn, and the content of α-tocopherol is 0.05 ± 0.00~0.16 ± 0.00 mg/100 g, the content of γ-tocopherol is approximately 0.23 ± 0.01~0.68 ± 0.05 mg/100 g, and δ-tocopherol is 0.02 ± 0.00~0.09 ± 0.04 (Table 11). It has good antioxidant activity, which can provide antioxidant protection for fresh sweet and waxy corn rich in unsaturated fatty acids, prevent the peroxidation reaction of corn during the processing and postprocessing storage stages, and maintain the nutritional value of fresh sweet and waxy corn.

The results of the factor analysis, comprehensive score calculation and ranking of the fatty acids in fresh corn showed that palmitic acid, oleic acid, and stearic acid, followed by linoleic acid, account for more than 62% of the index weight in the fatty acid composition of fresh corn, which proves that these fatty acids were of great significance in the process of the nutritional composition and flavour formation of fresh corn. Oleic acid can stimulate human bile secretion and promote fat digestion [78]. Arachidonic acid and palmitic acid have a significant effect on human body composition and nutritional reserves. The comprehensive score of the factor analysis was fresh corn 2-4 > 2-2 > 2-1 > 2-4 > 3-3 > 1-2 > 3-2 > 1-1 > 1-4 > 1-3 > 3-1 > 1-5, but the rank result was only based on factor analysis results; it could not represent the ranking of the fatty acid quality of maize in the three regions because the evaluation of fatty acids is complex. 

The fatty acid oxidation properties compared with other places are defects, which should be considered in the process of processing and storage. The content of tocopherol in fresh maize 1-4 in Inner Mongolia was the highest, and the value of ω-6:ω-3 was the second lowest, which was closest to the recommended value, suggesting that the essential fatty acid composition of fresh maize 1-4 was one of the most suitable foods to eat for the dietary supplementation of essential fatty acids.

## 4. Conclusions

In this study, the contents and composition profiles of fatty acids in fresh sweet-waxy maize were detected and analysed. The results showed that the fatty acid content decreased in the order linoleic acid > oleic acid > palmitic acid > α-linolenic acid > stearic acid, from high to low. The UFA content in fresh sweet-waxy corn produced is more than 70% of the total fatty acid content. The linoleic acid and α-linolenic acid contents are rich, and fatty acid composition and content differ between the different regions: seven kinds of fatty acids in fresh sweet-waxy corn 3-3 were detected, then the fresh corn 1-3 showed 11 fatty acids, and fresh corns 1-5, 3-1 and 3-2 showed 12 fatty acids. Then, 13 fatty acids were detected in fresh corns 1-1, 1-2, and 1-4 from Inner Mongolia, and 14 kinds of fatty acids were detected in fresh corn 2-4. Fresh corns 2-1, 2-2, and 2-3 had the most various fatty acids (15 kinds of fatty acids). 

The experimental results showed that fresh corn is rich in UFAs and has low-atherogenic, low-thrombogenic, and high oxidation sensitivity nutritional characteristics. According to the results of the factor analysis and index evaluation, the fatty acid content and factor analysis comprehensive score of fresh corn 2-4 in the Jilin region were the highest, while the atherosclerosis index and thrombosis index were lower, and the comprehensive quality of fresh corn was the best. The contents of essential fatty acids and γ-tocopherol of fresh maize 3-1 from Heilongjiang province were higher than those from the other regions. The quality of fresh maize in Heilongjiang is better than that in other regions in terms of supplementing the essential fatty acids and antioxidant properties of fatty acids. The composition of unsaturated fatty acids in the total fatty acids of fresh corns 3-1, 3-2, 3-3 in Heilongjiang Province is higher than those of the other samples, and the index of atherosclerosis and thrombosis is the lowest, which has a great advantage in the prevention and treatment of cardiovascular and cerebrovascular diseases.

Different people are fitted with different dietary patterns. In this article, we evaluated 12 samples to support the following dietary recommendation: people who need more kinds of fatty acids are recommended to eat fresh corns 2-1, 2-2, 2-3 and 2-4 from Jilin province; the people who would like to take in more essential fatty acids and tocopherols are recommended to eat fresh corn from Heilongjiang Province.

As an important part of the nutritional quality evaluation of fresh corn, fatty acid evaluation can provide scientific and effective evidence to verify the nutritional value of fresh corn as a new staple food. Fresh corn tastes fresh and tender, and its market share increases year by year. On the one hand, its processing and storage technology is advanced, which can maximize the retention of natural UFAs and other nutrients in fresh corn. On the other hand, because the market price of fresh corn is higher than that of ordinary corn, planting fresh corn brings higher economic benefits under the field planting mode, which can increase farmers’ income. In recent years, fresh corn breeding and planting techniques for long-term development have provided a good technical means for industry development. Sweet-waxy corn, as the latest achievement of fresh corn hybrid breeding, has a combination of sweet corn and waxy maize with good sensory qualities and nutritional value; in particular, the fatty acid composition is greater than that of other staples, such as wheat and rice, and is in line with human needs. It can be used as a daily staple food to supplement dietary fatty acids for all populations to adjust the composition and proportion of fatty acid intake in their daily diet.

## Figures and Tables

**Table 1:** Linear regression equations, correlation coefficients, LOD, LOQ, instrument accuracy, and recovery of fatty acids.

Fatty Acids	Linear Regression Equations	Correlation Coefficients (R^2)^	Limit of Detection (g/100 g)	Limit of Quantitation (mg/L)	RSD (%)	Recovery (%)
C10:0	Y = 0.0052X − 0.087	0.9991	0.01	0.0033	2.9	89.1
C12:0	Y = 0.0034X − 0.085	0.9998	0.01	0.0033	3.3	93.2
C14:0	Y = 0.0033X − 0.023	0.9993	0.01	0.0033	3.4	89.9
C16:0	Y = 0.0032X − 0.065	0.9995	0.01	0.0033	3.5	101.2
C16:1n7	Y = 0.0046X − 0.054	0.9999	0.01	0.0033	5.1	94.3
C17:0	Y = 0.0015X − 0.072	0.9998	0.01	0.0033	4.9	102.5
C18:0	Y = 0.0007X − 0.095	0.9995	0.01	0.0033	5.3	93.3
C18:1n9c	Y = 0.0075X − 0.024	0.9997	0.01	0.0033	4.8	90.0
C18:2n6c	Y = 0.0025X + 0.008	0.9998	0.01	0.0033	3.7	89.7
C20:0	Y = 0.0043X − 0.081	0.9995	0.01	0.0033	3.1	95.5
C20:1	Y = 0.0023X − 0.062	0.9992	0.01	0.0033	2.6	95.3
C18:3n3	Y = 0.0026X + 0.006	0.9995	0.01	0.0033	6.5	112.3
C22:0	Y = 0.0034X − 0.029	0.9993	0.01	0.0033	6.0	96.7
C23:0	Y = 0.0048X − 0.082	0.9997	0.01	0.0033	4.2	96.4
C24:0	Y = 0.0019X − 0.030	0.9999	0.01	0.0033	5.3	105.0
C20:5n3	Y = 0.0012X − 0.035	0.9996	0.01	0.0033	3.4	99.5

**Table 2:** Linear regression equations, correlation coefficients, LOD, LOQ, instrument accuracy, and recovery of tocopherols.

Tocopherols	Linear Regression Equations	Correlation Coefficients (R^2)^	Limit of Detection (g/100 g)	Limit of Quantitation (mg/L)	RSD (%)	Recovery (%)
α-tocopherol	Y = 0.0923X + 4.63	0.9996	0.4	0.12	2.74	100.5
γ-tocopherol	Y = 0.0034X − 0.085	0.9993	0.4	0.12	0.83	98.9
δ-tocopherol	Y = 0.0033X − 0.023	0.9999	0.4	0.12	1.35	97.4

**Table 3:** Relative fatty acid contents of fresh sweet-waxy corn from the three producing provinces (% of total fatty acids).

	1-1	1-2	1-3	1-4	1-5	2-1	2-2	2-3	2-4	3-1	3-2	3-3
C10:0	-	-	-	-	-	0.07 ± 0.01	0.07 ± 0.02	0.09 ± 0.02	0.05 ± 0.01	-	-	-
C12:0	0.18 ± 0.15	0.05 ± 0.00	0.06 ± 0.01	0.07 ± 0.01	0.23 ± 0.15	0.03 ± 0.00	0.06 ± 0.02	0.05 ± 0.01	0.06 ± 0.02	0.09 ± 0.03	0.07 ± 0.00	-
C14:0	0.24 ± 0.25	0.08 ± 0.01	-	0.11 ± 0.01	0.14 ± 0.05	0.05 ± 0.00	0.07 ± 0.02	0.06 ± 0.00	0.06 ± 0.00	0.08 ± 0.02	0.04 ± 0.01	-
C16:0	18.90 ± 0.50 ^ef^	18.04 ± 0.58 ^d^	18.34 ± 0.31 ^f^	18.97 ± 0.85 ^de^	19.50 ± 0.12 ^h^	17.20 ± 1.00 ^b^	15.67 ± 0.19 ^bc^	16.65 ± 0.18 ^b^	17.25 ± 0.04 ^a^	13.48 ± 0.14 ^g^	16.36 ± 0.20 ^de^	14.87 ± 0.67 ^c^
C16:1n7	0.19 ± 0.00 ^c^	0.17 ± 0.01 ^c^	0.16 ± 0.03 ^cd^	0.19 ± 0.05 ^c^	0.25 ± 0.02 ^cd^	0.20 ± 0.04 ^ab^	0.17 ± 0.01 ^b^	0.19 ± 0.00 ^ab^	0.20 ± 0.01 ^a^	0.11 ± 0.01 ^d^	0.09 ± 0.00 ^d^	0.13 ± 0.00 ^c^
C17:0	0.10 ± 0.01	0.14 ± 0.02	0.15 ± 0.02	0.10 ± 0.03	-	0.22 ± 0.00	0.19 ± 0.00	0.23 ± 0.01	0.06 ± 0.09	0.09 ± 0.01	0.14 ± 0.02	-
C18:0	1.75 ± 0.19 ^fg^	1.81 ± 0.01 ^de^	1.55 ± 0.06 ^gh^	1.93 ± 0.20 ^de^	1.55 ± 0.08 ^i^	2.47 ± 0.10 ^b^	2.62 ± 0.03 ^a^	2.29 ± 0.06 ^c^	2.06 ± 0.02 ^c^	1.39 ± 0.01 ^h^	1.52 ± 0.03 ^ef^	1.38 ± 0.09 ^d^
C18:1 n9c	24.91 ± 0.45 ^de^	28.56 ± 1.22 ^bc^	24.89 ± 0.87 ^de^	25.60 ± 1.84 ^de^	20.48 ± 0.08 ^f^	36.66 ± 0.12 ^a^	36.62 ± 0.11 ^a^	35.82 ± 0.15 ^a^	34.81 ± 0.14 ^a^	21.04 ± 0.25 ^e^	23.33 ± 0.40 ^cd^	22.39 ± 0.26 ^b^
C18:2n6c	50.08 ± 0.61 ^e^	46.76 ± 0.34 ^de^	51.22 ± 0.25 ^e^	49.10 ± 0.32 ^de^	53.21 ± 1.00 ^f^	40.51 ± 1.12 ^cd^	42.06 ± 0.09 ^bc^	42.11 ± 0.01 ^bc^	43.30 ± 0.15 ^b^	61.25 ± 0.09 ^bc^	55.73 ± 0.02 ^b^	59.48 ± 0.59 ^a^
C20:0	0.50 ± 0.03 ^c^	0.53 ± 0.04 ^b^	0.49 ± 0.02 ^c^	0.27 ± 0.02 ^e^	0.56 ± 0.09 ^de^	0.42 ± 0.02 ^ab^	0.45 ± 0.01 ^ac^	0.43 ± 0.00 ^ab^	0.42 ± 0.01 ^ab^	0.27 ± 0.00 ^e^	0.36 ± 0.01 ^d^	0.29 ± 0.01 ^cd^
C20:1	0.31 ± 0.01 ^b^	0.31 ± 0.01 ^b^	0.33 ± 0.03 ^b^	0.29 ± 0.02 ^b^	0.32 ± 0.12 ^c^	0.23 ± 0.00 ^b^	0.23 ± 0.00 ^b^	0.24 ± 0.01 ^b^	0.21 ± 0.01 ^b^	0.17 ± 0.02 ^c^	0.12 ± 0.00 ^c^	1.46 ± 0.08 ^a^
C18:3 n3	2.40 ± 0.00	2.41 ± 0.17	2.52 ± 0.22	2.69 ± 0.39	3.06 ± 0.09	1.21 ± 0.08	1.09 ± 0.02	1.20 ± 0.03	1.25 ± 0.02	1.89 ± 0.09	1.97 ± 0.03	-
C22:0	0.30 ± 0.04	0.28 ± 0.02	0.29 ± 0.02	0.27 ± 0.02	0.54 ± 0.01	0.12 ± 0.00	0.13 ± 0.00	0.14 ± 0.02	0.16 ± 0.00	0.15 ± 0.05	0.27 ± 0.12	-
C23:0	-	-	-	-	-	0.13 ± 0.01	0.11 ± 0.01	0.14 ± 0.02	0.09 ± 0.01	-	-	-
C24:0	-	0.86 ± 0.03	-	-	-	0.49 ± 0.02	0.48 ± 0.07	0.40 ± 0.04	-	-	-	-
C20:5n3	0.15 ± 0.01	-	-	0.16 ± 0.01	0.26 ± 0.07	-	-	-	-	-	-	-
Total fatty acids content (g/100 g)	1.24 ± 0.07 ^cd^	1.42 ± 0.07 ^bc^	1.23 ± 0.06 ^d^	1.32 ± 0.02 ^cd^	0.81 ± 0.00 ^e^	1.83 ± 0.10 ^a^	1.93 ± 0.06 ^a^	1.85 ± 0.07 ^a^	1.99 ± 0.12 ^a^	1.33 ± 0.09 ^cd^	1.54 ± 0.11 ^b^	1.91 ± 0.09 ^a^

Notes: Each datum is an average with three parallel samples, with standard deviations. - indicates that the fatty acid was not detected, or that the content of fatty acid was below the detection limit of 0.003 g/100 g. ^a–h^ represents significant differences in fatty acid content in different regions at the 0.05 level.

**Table 4:** Fatty acid composition analysis of fresh sweet-waxy corn from different producing areas.

	1-1	1-2	1-3	1-4	1-5	2-1	2-2	2-3	2-4	3-1	3-2	3-3
SFA(%)	21.97 ± 1.09	21.79 ± 0.69	20.88 ± 0.41	21.82 ± 1.05	22.52 ± 0.48	21.20 ± 1.12	19.83 ± 0.20	20.49 ± 0.21	20.22 ± 0.01	15.55 ± 0.22	18.76 ± 0.35	16.54 ± 0.76
MUFA(%)	25.41 ± 0.46	29.04 ± 1.19	25.38 ± 0.87	26.08 ± 1.77	21.05 ± 0.06	37.09 ± 0.08	37.02 ± 0.12	36.24 ± 0.16	35.23 ± 0.14	21.31 ± 0.22	23.55 ± 0.40	23.98 ± 0.18
PUFA(%)	52.63 ± 0.63	49.17 ± 0.50	53.74 ± 0.46	51.95 ± 0.71	56.53 ± 1.16	41.72 ± 1.04	43.15 ± 0.07	43.31 ± 0.04	44.56 ± 0.13	63.14 ± 0.00	57.69 ± 0.05	59.48 ± 0.59
UFA(%)	78.03 ± 1.09	78.21 ± 0.69	79.12 ± 0.41	78.03 ± 1.06	77.57 ± 1.10	78.80 ± 1.12	80.17 ± 0.20	79.54 ± 0.19	79.78 ± 0.01	84.45 ± 0.22	81.24 ± 0.35	83.46 ± 0.76
DFA(%)	79.78 ± 0.90	80.02 ± 0.70	80.68 ± 0.35	79.96 ± 0.86	79.12 ± 1.17	81.27 ± 1.02	82.78 ± 0.17	81.84 ± 0.13	81.84 ± 0.03	85.83 ± 0.23	82.76 ± 0.32	84.84 ± 0.68
n-6:n-3	19.66 ± 0.16	19.42 ± 1.21	20.38 ± 1.65	17.37 ± 2.30	16.04 ± 0.49	33.64 ± 3.18	38.6 ± 0.77	35.14 ± 0.74	34.63 ± 0.75	32.46 ± 1.60	28.33 ± 0.42	-
SFA:MUFA:PUFA	1:1.2:2.4	1:1.3:2.3	1:1.2:2.6	1:1.2:2.4	1:0.9:2.5	1:1.7:2	1:1.9:2.2	1:1.8:2.1	1:1.7:2.2	1:1.4:4.1	1:1.3:3.1	1:1.4:3.6

Notes: Each datum is an average with three parallel samples, with standard deviations. SFAs: saturated fatty acids; UFAs: unsaturated fatty acids; MUFAs: monounsaturated fatty acids; PUFAs: polyunsaturated fatty acids; DFAs: UFA + C18:0; -: C18:3n3 was not detected in fresh corn 3-3.

**Table 5:** Correlation of the fatty acid composition in sweet-waxy corn.

	C16:0	C16:1n7	C18:0	C18:1n9c	C18:2n6c	C20:0	C20:1
C16:0	1						
C16:1n7	0.821 **	1					
C18:0	0.876 **	0.841 **	1				
C18:1n9c	0.921 **	0.842 **	0.976 **	1			
C18:2n6c	0.544 **	0.141	0.367	0.448 *	1		
C20:0	0.776 **	0.730 **	0.791 **	0.834 **	0.210	1	
C20:1	0.221	0.037	0.002	0.053	0.685 **	−0.035	1

Notes: ** represents a significant correlation between fatty acids at the 0.01 level. * represents a significant correlation between fatty acids at the 0.05 level.

**Table 6:** Fatty acid characteristic value and the variance contribution rate of fresh sweet-waxy corn in the regions.

Principal Component	Initial Eigenvalue			Rotate Square and Load		
	Eigenvalue	Variance Contribution Rate%	Cumulative Variance Contribution Rate%	Eigenvalue	Rotate Variance Contribution Rate%	Cumulative Rotate Variance Contribution Rate%
PC1	4.562	65.171	65.171	4.377	62.535	62.535
PC2	1.621	23.156	88.327	1.805	25.792	88.327

**Table 7:** Analysis of the fatty acid factor of fresh sweet-waxy maize by the rotating component matrix.

Fatty Acids	Principle Components	
	PC1	PC2
C16:0	0.908	0.327
C16:1n7	0.906	−0.008
C18:0	0.959	0.097
C18:1n9c	0.970	0.168
C18:2n6c	0.269	0.899
C20:0	0.890	−0.017
C20:1	−0.065	0.923

**Table 8:** Weight of different fatty acids in the quality evaluation of fresh sweet-waxy corn.

Fatty Acids	Index Weight
C16:0	0.169
C16:1n7	0.130
C18:0	0.148
C18:1n9c	0.158
C18:2n6c	0.146
C20:0	0.129
C20:1	0.120

**Table 9:** Comprehensive score (F) and ranking of the fatty acid quality evaluation of fresh maize in three regions.

Regions	PC1 Score (Y1)	PC2 Score (Y2)	Synthesis Score (F)	Rank
1-1	−0.406	- 0.525	−0.440	8
1-2	0.030	−0.399	−0.095	6
1-3	−0.613	−0.433	−0.560	10
1-4	−0.529	−0.300	−0.465	9
1-5	−1.281	−1.186	−1.255	12
2-1	1.245	−0.266	0.800	3
2-2	1.343	−0.100	0.920	2
2-3	1.137	−0.141	0.765	4
2-4	1.441	0.092	1.045	1
3-1	−1.276	0.055	−0.885	11
3-2	−0.582	0.184	−0.355	7
3-3	−0.509	3.020	0.520	5

**Table 10:** Fatty acid evaluation indices of fresh sweet-waxy corn in the three regions.

	1-1	1-2	1-3	1-4	1-5	2-1	2-2	2-3	2-4	3-1	3-2	3-3
AI	0.32 ± 0.01 ^c^	0.33 ± 0.00 ^c^	0.29 ± 0.01 ^d^	0.33 ± 0.01 ^c^	0.21 ± 0.00 ^e^	0.40 ± 0.01 ^b^	0.38 ± 0.00 ^b^	0.39 ± 0.01 ^b^	0.44 ± 0.03 ^a^	0.22 ± 0.01 ^e^	0.31 ± 0.02 ^c^	0.34 ± 0.00 ^c^
TI	0.01 ± 0.00	0.01 ± 0.00	0.01 ± 0.00	0.02 ± 0.00	0.01 ± 0.00	0.01 ± 0.00	0.01 ± 0.00	0.01 ± 0.00	0.01 ± 0.00	0.01 ± 0.00	0.01 ± 0.00	-
HPI	3.14 ± 0.13 ^c^	2.99 ± 0.02 ^c^	3.49 ± 0.10 ^b^	3.03 ± 0.12 ^c^	4.72 ± 0.05 ^a^	2.47 ± 0.05 ^de^	2.60 ± 0.02 ^d^	2.54 ± 0.06 ^d^	2.28 ± 0.13 ^e^	4.58 ± 0.21 ^a^	3.18 ± 0.18 ^c^	2.95 ± 0.02 ^c^
PI	0.70 ± 0.05 ^e^	0.74 ± 0.03 ^de^	0.70 ± 0.03 ^e^	0.74 ± 0.00 ^de^	0.50 ± 0.01 ^f^	0.80 ± 0.06 ^cde^	0.87 ± 0.03 ^bc^	0.84 ± 0.03 ^bcd^	0.93 ± 0.06 ^b^	0.87 ± 0.06 ^bc^	0.93 ± 0.06 ^b^	1.15 ± 0.07 ^a^
h/H	3.49 ± 0.09 ^defg^	3.48 ± 0.07 ^efg^	3.72 ± 0.03 ^cd^	3.39 ± 0.18 ^fg^	4.55 ± 0.00 ^b^	3.45 ± 0.17 ^fg^	3.74 ± 0.02 ^c^	3.53 ± 0.00 ^cdefg^	3.29 ± 0.08 ^g^	5.03 ± 0.14 ^a^	3.70 ± 0.10 ^cde^	3.61 ± 0.10 ^cdef^
HVI	1.41 ± 0.05 ^e^	1.69 ± 0.12 ^c^	1.44 ± 0.07 ^de^	1.45 ± 0.15 ^de^	1.13 ± 0.00 ^f^	2.28 ± 0.13 ^b^	2.50 ± 0.04 ^a^	2.29 ± 0.03 ^b^	2.14 ± 0.00 ^b^	1.66 ± 0.04 ^c^	1.52 ± 0.04 ^cde^	1.60 ± 0.08 ^cd^

Notes: Each datum is an average with three parallel samples, with standard deviations. ^a–g^ represents significant differences of same indicator among different samples at the level of 0.05.

**Table 11:** Content of tocopherols in fresh corn in the three regions.

	1-1	1-2	1-3	1-4	1-5	2-1	2-2	2-3	2-4	3-1	3-2	3-3
α-tocopherol	0.08 ± 0.02	0.07 ± 0.02	0.09 ± 0.02	0.07 ± 0.00	0.05 ± 0.00	0.16 ± 0.00	0.07 ± 0.01	-	-	-	-	-
γ-tocopherol	0.54 ± 0.04 ^b^	0.62 ± 0.04 ^a^	0.46 ± 0.02 ^c^	0.68 ± 0.05 ^a^	0.23 ± 0.01 ^e^	0.44 ± 0.01 ^c^	0.47 ± 0.03 ^c^	0.32 ± 0.02 ^d^	0.30 ± 0.01 ^d^	0.44 ± 0.02 ^c^	0.31 ± 0.00 ^d^	0.48 ± 0.03 ^bc^
δ-tocopherol	0.09 ± 0.00 ^a^	0.09 ± 0.04 ^a^	0.07 ± 0.02 ^ab^	0.05 ± 0.01 ^bc^	0.02 ± 0.01 ^c^	0.04 ± 0.00 ^bc^	0.04 ± 0.00 ^c^	0.03 ± 0.00 ^c^	0.03 ± 0.00 ^c^	0.02 ± 0.00 ^c^	0.01 ± 0.00 ^c^	0.02 ± 0.01 ^c^

Notes: Each data was an average with three parallel samples, with it standard deviations. ^a–d^ represents significant differences in the tocopherol content of fresh corn (*p* < 0.05).

## Data Availability

The data presented in this study are available on request from the corresponding author. The data are not publicly available due to its small scale.

## Abbreviations

AI	atherosclerosis index
BHT	2,6-butylated hydroxytoluene
DFA	ideal fat index
EPA	eicosapentaenoic acid
FAME	fatty acid methyl ester
FAQI	fatty acid quality index
GC	gas chromatography
H/h	fatty acid ratio of hypocholesterolemia to hypercholesterolemia
HPI	health promotion index
MUFAs	monounsaturated fatty acids
NVI	nutritional value index
PC	principal component
PCA	principal components analysis
PUFAs	polyunsaturated fatty acids
SFAs	saturated fatty acids
TI	thrombosis index
UFAs	unsaturated fatty acids
WHO	World Health Organization
